# Predictors of pulmonary metastases on chest computed tomography in children and adolescents with osteosarcoma—tips for qualifying patients for thoracotomy

**DOI:** 10.1186/s12887-024-04858-0

**Published:** 2024-06-03

**Authors:** Marek Duczkowski, Agnieszka Duczkowska, Anna Olwert, Elżbieta Michalak, Katarzyna Bilska, Teresa Klepacka, Magdalena Rychłowska-Pruszyńska, Anna Raciborska, Monika Bekiesińska-Figatowska

**Affiliations:** 1 Department of Diagnostic Imaging, Institute of Mother and Child, Warsaw, 01-211 Poland; 2 WIT Academy, Warsaw, 01-447 Poland; 3 Department of Pathomorphology, Institute of Mother and Child, Warsaw, 01-211 Poland; 4 Department of Oncology and Surgical Oncology for Children and Youth, Institute of Mother and Child, Warsaw, 01-211 Poland

**Keywords:** Osteosarcoma, Pulmonary metastases, Computed tomography, Thoracotomy, Metastasectomy, Children, Predictor

## Abstract

**Background:**

Osteosarcoma is the most common primary malignant bone tumour in children and adolescents. Lungs are the most frequent and often the only site of metastatic disease. The presence of pulmonary metastases is a significant unfavourable prognostic factor. Thoracotomy is strongly recommended in these patients, while computed tomography (CT) remains the gold imaging standard. The purpose of our study was to create tools for the CT-based qualification for thoracotomy in osteosarcoma patients in order to reduce the rate of useless thoracotomies.

**Methods:**

Sixty-four osteosarcoma paediatric patients suspected of lung metastases on CT and their first-time thoracotomies (*n* = 100) were included in this retrospective analysis. All CT scans were analysed using a compartmental evaluation method based on the number and size of nodules. Calcification and location of lung lesions were also analysed. Inter-observer reliability between two experienced radiologists was assessed. The CT findings were then correlated with the histopathological results of thoracotomies. Various multivariate predictive models (logistic regression, classification tree and random forest) were built and predictors of lung metastases were identified.

**Results:**

All applied models proved that calcified nodules on the preoperative CT scan best predict the presence of pulmonary metastases. The rating of the operated lung on the preoperative CT scan, dependent on the number and size of nodules, and the total number of nodules on this scan were also found to be important predictors. All three models achieved a relatively high sensitivity (72–92%), positive predictive value (81–90%) and accuracy (74–79%). The positive predictive value of each model was higher than of the qualification for thoracotomy performed at the time of treatment. Inter-observer reliability was at least substantial for qualitative variables and excellent for quantitative variables.

**Conclusions:**

The multivariate models built and tested in our study may be useful in the qualification of osteosarcoma patients for metastasectomy through thoracotomy and may contribute to reducing the rate of unnecessary invasive procedures in the future.

## Background

Osteosarcoma (OS) is the most common primary malignant bone tumour in children and adolescents [1]. OS is characterised by rapid hematogenous spread, with the lung being the most common site [2–4]. According to the literature, 10–25% of OS patients present with detectable metastases at the time of initial diagnosis, of which 85–90% have lung metastases [1, 5–9]. Tumour cells in OS metastases produce bone and this potential may be apparent on imaging. Moreover, the recurrence of OS is predominantly located in the lung (~ 80% of cases) [1]. For the assessment of pulmonary metastases, chest computed tomography (CT) has remained for years the gold imaging standard, as also suggested by the Children’s Oncology Group (COG) [6, 10–12]. With the continuous advances in multi-row-detector computed tomography (MDCT) scanners, the sensitivity in detecting small lung nodules has improved. Nonetheless, the distinction between malignant and benign pulmonary lesions on CT scans in paediatric patients with sarcomas is still below expectations [6, 11, 13–15], and the correct classification of pulmonary nodules on imaging as either malignant or benign is a clinical dilemma even for radiologists experienced in the field.

The presence of metastases has a significant impact on survival in OS patients [2, 6, 16–18]. Therefore, early diagnosis and appropriate treatment is an interdisciplinary challenge for the entire team involved, including the oncologist, surgeon, radiologist and pathologist. All OS metastases must be resected completely, regardless of their number and site, if the patient is treated with curative intent [5, 8, 19–22]. For pulmonary metastases, thoracotomy (TT) with manual exploration of the lung, which may improve survival, is the most strongly recommended technique and should be considered whenever feasible, even when repeated procedures are required [1, 5, 23–26].

On the other hand, TT is an additional burden for the oncologic patient. Moreover, a substantial percentage of paediatric patients with malignant solid primary tumours undergo invasive thoracic procedures that reveal only benign nodules [13, 14, 27]. We therefore assumed that a proper radiological qualification of OS patients for TT is highly significant. While both in the literature and in multi-institutional clinical trial protocols various criteria of lung metastases have been proposed [5, 13, 15, 27–29], to date no CT-based guidelines for qualifying patients with OS for TT have been established.

The purpose of this study was to construct multivariate models revealing tomographic predictors of lung metastases in children and adolescents with OS. An appropriate model will predict which TTs are justified (by confirming the presence of at least one pulmonary metastasis) and which are not. This in turn may contribute to reducing the rate of useless TTs.

## Methods

**Fig. 1:**
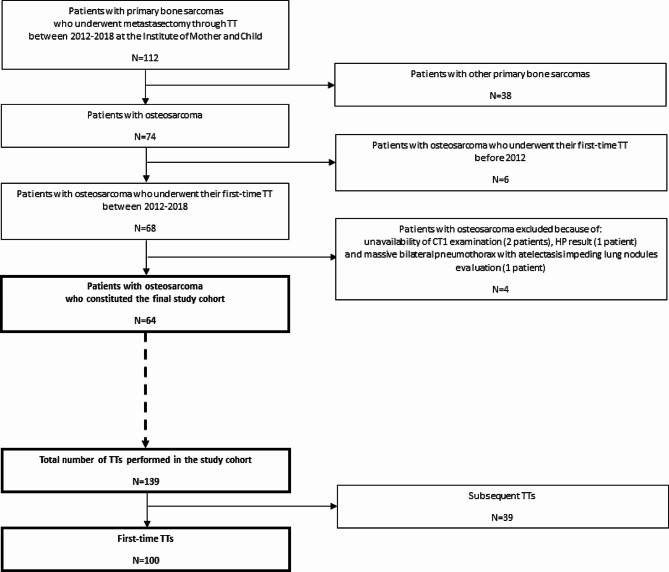
Study flow chart - osteosarcoma patients and their thoracotomies

This retrospective study was approved by the Bioethics Committee of the Institute of Mother and Child in Warsaw, Poland, with a waiver of parental and patient informed consent. The final study cohort was constituted by sixty-four high-grade OS paediatric patients, who were diagnosed and treated in accordance with the trial protocol of the European and American Osteosarcoma Study Group (EURAMOS) 1 [5]. They were selected from a larger group of patients with primary bone sarcomas (*n* = 112), after excluding patients with other bone sarcomas and patients with osteosarcoma who did not fulfil our inclusion criteria. All included osteosarcoma patients underwent their first-time metastasectomy through TT for presumed pulmonary metastases on chest CT between 2012 and 2018. Out of the total 139 first-time and subsequent TTs performed during the 7-year time period, only 100 first-time procedures (right- and/or left-sided) were included in the study, i.e., one or maximum two TTs per patient, in order to eliminate the influence of postoperative sequelae on CT evaluation. Figure 1 illustrates the study flow chart. In bilateral lesions, staged surgery was performed. Manual palpation of the lung was carried out through lateral TT and wedge resections were performed whenever possible. All lesions removed were studied histologically and the results are available in the archives of the Department of Pathomorphology. For each included TT, we reviewed two spiral chest CT scans queried on our picture archiving and communication system (PACS): the baseline CT scan (CT1), i.e., the first examination which revealed lung nodules on the eventually operated side, and the preoperative CT scan (CT2), i.e., the examination directly preceding the TT. The CT1 scan was performed before the start of treatment (for patients suspected of pulmonary metastases at diagnosis), during treatment (for patients suspected of disease progression), or during follow-up after treatment (for patients suspected of pulmonary recurrence). Nearly all CT scans were performed on MDCT scanners, of which most on Philips Brilliance 64 (64-detector row) with iDose reconstruction algorithms. Intravenous contrast was not administered routinely.

CT scans were retrospectively and independently reviewed by two radiologists with 15 years of experience at our institution (the national reference centre for children with bone and soft tissue tumours) who were blinded to the outcome of each TT. All available tools, including multiplanar reformatted reconstructions (MPR) and the maximum intensity projection (MIP) technique, were used on a dedicated workstation (Philips Extended Brilliance Workspace). Reader 1 reviewed CT scans for all included TTs (*n* = 100), and his findings were then correlated with the histopathological (HP) status of the TTs. Reader 2 reviewed CT scans for 50 randomly selected TTs, for which the inter-observer reliability between the two readers was tested. On all CT1 scans, both lungs together as well as the operated lung separately were assessed, while on the CT2 scans, the operated lung was assessed once again, each time using a compartmental evaluation method based on the number of nodules and their maximum axial diameter. For the combined assessment of both lungs, scoring criteria were adopted from the EURAMOS-1 protocol: “A”—at least one nodule of ≥ 10 mm or three or more nodules of ≥ 5 mm; “B”—scans not meeting score “A” criteria. We applied the following rating criteria for the operated lung: “a”—single nodule of > 10 mm or more than one nodule of > 5 mm; “b”—solitary nodule of 5–10 mm or multiple nodules of 3–5 mm; “c”—solitary nodule of < 5 mm or several nodules of < 3 mm. On both CT1 and CT2 scans, we also recorded the presence of calcified nodules, while on CT2 scans the total number of nodules, their maximum axial diameter and their location, which was determined using the depth from the nearest pleural surface (< 10 mm = peripheral including subpleural and pleural; ≥10 mm = central), were also noted.

HP status was presented on a three-point scale (0-1-2), where “0” was the presence of solely nonmetastatic nodules in the resected material, “1”—at most nonviable metastases (minimum 1 lesion of complete regression), and “2”—at least 1 viable metastasis.

An analysis of CT1 and CT2 characteristics was performed in order to find variables differentiating the two TT samples: with confirmed metastases (HP group “1 + 2”) and without metastases (HP group “0”). For qualitative variables, the two-sample z-test for proportions was used, while for quantitative variables, the Wilcoxon rank sum test was used. Three various multivariate predictive models were constructed (logistic regression, decision tree and random forest) and predictors of the HP status of TTs were identified. To examine which features were associated with TT outcome in the logistic regression, a multivariate model was created in which all predictors with univariate p values of < 0.20 were entered into the model. The model was reduced until only those predictors with p values of < 0.05 (considered statistically significant) remained. In the decision tree and random forest models predictors were selected by the algorithms used in these models. Each model was tested for sensitivity, specificity, positive predictive value (PPV), negative predictive value (NPV) and accuracy, using leave-one-out cross-validation (for the logistic regression and decision tree models) and the bootstrap method (for the random forest model). The PPV of the models was compared with the PPV of the qualification for TT at the time of the treatment of the analysed patients. To assess the inter-observer reliability, Cohen’s kappa coefficient (κ) for qualitative variables and Lin’s concordance correlation coefficient (rc) for quantitative variables were used. Pearson’s correlation coefficient (r) was used to test the correlation between the quantitative variables. Statistical analyses were performed using the R software environment (version 4.0.5) under the GNU General Public License.

## Results

### Patient and TT characteristics

Our cohort consisted of 64 patients with OS, 46 (72%) males and 18 (28%) females, with the primary tumour predominantly located in the peripheral skeleton: 34 (53%) in the femur, 15 (23%) in the tibia and 9 (14%) in the humerus; the mean age at the time of the biopsy of the primary tumour was 13.7 years (range: 5–19 years). Among their 100 first-time TTs included in this study, there were 72 staged bilateral metastasectomies (in 36 patients) and 28 unilateral metastasectomies, with 55 right-sided and 45 left-sided procedures. The mean time from the initial diagnosis to the TT was 12.9 months (range: 3–133 months). Table 1 presents the TT characteristics.

**Table 1:** Thoracotomy characteristics

Characteristic	Distribution
Laterality	
Bilateral (*n*, %)	72 (72.0%)
Unilateral (*n*, %)	28 (28.0%)
Operated lung	
Right (*n*, %)	55 (55.0%)
Left (*n*, %)	45 (45.0%)
Time from diagnosis to TT (months)	
Mean ± SD	12.9 ± 15.83
Min– median–max	3.6–7.8–133.2

TT: thoracotomy; SD: standard deviation; *n*: number

### CT1 scans

The combined rating of both lungs was “B” for 73 (73%) TTs, while the score of the operated lung only was “b” for 43 (43%) TTs. Calcified nodules were recorded on CT1 scans of 41 (41%) TTs. The mean time from the CT1 scan to the TT was 7.5 months (range: 1–33 months). Table 2 presents the characteristics of the CT1 scans.

**Table 2:** Characteristics of CT1 scans

Variable	Distribution
Combined rating of both lungs	
A (*n*, %)	27 (27%)
B (*n*, %)	73 (73%)
Rating of the operated lung	
a (*n*, %)	26 (26%)
b (*n*, %)	43 (43%)
c (*n*, %)	31 (31%)
Calcified nodules	
Present (*n*, %)	41 (41%)
Absent (*n*, %)	52 (52%)
n/a (*n*, %)	7 (7%)
Time from CT1 scan to TT (months)	
Mean ± SD	7.5 ± 4.41
Min– median–max	0.95–6.3–33.2

CT1: baseline computed tomography scan; TT: thoracotomy; A—at least one nodule of ≥ 10 mm or three or more nodules of ≥ 5 mm; B—scans not meeting score A criteria; a—single nodule of > 10 mm or more than one nodule of > 5 mm; b—solitary nodule of 5–10 mm or multiple nodules of 3–5 mm; c—solitary nodule of < 5 mm or several nodules of < 3 mm; SD: standard deviation; n/a: not applicable; *n*: number

### CT2 scans

The total number of nodules detected and analysed on all CT2 scans was 630, while the mean number of nodules per TT was 6.3 (range: 1–32). The score of the operated lung was “b” for 51 (51%) TTs. Calcified nodules were found in 58 (58%) TTs; the mean number of calcified nodules was 1.3 (range: 0–10). Most lesions were located peripherally (mean 5.1, of which 2.2 sub-/pleurally). The mean time from the CT2 scan to the TT was 13.9 days (range: 0–84 days); the median was 7.5 days. Table 3 presents the characteristics of the CT2 scans.

**Table 3:** Characteristics of CT2 scans

Variable	Distribution
Total number of nodules per TT	
Mean ± SD	6.3 ± 5.90
Min– median–max	1–4.5–32
Number of nodules of < 3.0 mm	
Mean ± SD	3.25 ± 3.38
Min– median–max	0–2–18
Number of nodules of 3.0–5.0 mm	
Mean ± SD	2.13 ± 2.57
Min– median–max	0–1–14
Number of nodules of 5.1–10.0 mm	
Mean ± SD	0.64 ± 0.96
Min– median–max	0–0–5
Number of nodules of > 10.0 mm	
Mean ± SD	0.28 ± 0.71
Min– median–max	0–0–4
Rating of the operated lung	
a (*n*, %)	26 (26.0%)
b (*n*, %)	51 (51.0%)
c (*n*, %)	23 (23.0%)
Calcified nodules	
Present (*n*, %)	58 (58.0%)
Absent (*n*, %)	42 (42.0%)
Number of calcified nodules per TT	
Mean ± SD	1.31 ± 1.98
Min– median–max	0–1–10
Number of peripheral nodules per TT	
Mean ± SD	5.05 ± 4.94
Min– median–max	0–3–25
Number of central nodules per TT	
Mean ± SD	1.25 ± 1.65
Min– median–max	0–1–9
Time from CT2 scan to TT (days)	
Mean ± SD	13.9 ± 17.14
Min– median–max	0–7.5–84

CT2: preoperative computed tomography scan; TT: thoracotomy; a—single nodule of > 10 mm or more than one nodule of > 5 mm; b—solitary nodule of 5–10 mm or multiple nodules of 3–5 mm; c—solitary nodule of < 5 mm or several nodules of < 3 mm; SD: standard deviation; *n*: number

### HP outcome

The total number of nodules resected on all TTs was 422, while the mean number of nodules per TT was 4.2 (range: 1–19), of which 2.3 (54%) were nonmetastatic, 1.2 (29%) were viable metastases and 0.7 (17%) were nonviable metastases. The HP status of the TT was “2” for 54 (54%) TTs with at least 1 viable metastasis, “1” for 18 (18%) TTs with only nonviable metastases and “0” for 28 (28%) TTs confirming nonmetastatic nodules only. Among the latter, fibrosis, reactive changes, inflammation, atelectasis, intrapulmonary lymph nodes, congestion, haemorrhage, granulomas, normal lung parenchyma, focal pleural thickening and adhesions were the most common. Table 4 presents the HP outcome of the TTs.

**Table 4:** HP outcome of TTs

Variable	Distribution
Total number of nodules per TT	
Mean ± SD	4.2 ± 3.27
Min– median–max	1–3–19
Number of viable metastases	
Mean ± SD	1.22 ± 1.70
Min– median–max	0–1–8
Number of nonviable metastases	
Mean ± SD	0.73 ± 1.67
Min– median–max	0–0–10
Number of nonmetastatic nodules	
Mean ± SD	2.27 ± 2.06
Min– median–max	0–2–13
HP status of TT	
2—at least 1 viable metastasis (*n*, %)	54 (54.0%)
1—at most nonviable metastases (*n*, %)	18 (18.0%)
0—nonmetastatic nodules only (*n*, %)	28 (28.0%)

HP: histopathological; TT: thoracotomy; SD: standard deviation; *n*: number

In the two-sample z-test for proportions and the Wilcoxon rank sum test variables differentiating at a significance level of 0.05, two TT samples (HP group “1 + 2” and HP group “0”) were identified (Table 5). They include: the CT1 rating of both lungs (*p* = 0.04168 for score “A”), the presence of calcified nodules on the CT1 scan (*p* = 0.04276), the CT2 rating of the operated lung (*p* = 0.00334 for score “a”; *p* = 0.00019 for score “c”), the presence of calcified nodules on CT2 scan (*p* = 0.00001), the mean number of calcified nodules on the CT2 scan (*p* = 0.00005), the CT1-CT2 rating of the operated lung (*p* = 0.03011 for score “a-a”; *p* = 0.00026 for score “c-c”) and the mean number of nodules of > 10.0 mm on the CT2 scan (*p* = 0.00381).

**Table 5:** Analysis of variables differentiating thoracotomies with and without metastases

Variable	HP Group “0” (*n* = 28)	HP Group “1 + 2” (*n* = 72)	*p* Value
CT1 score of both lungs			
A (*n*, %)	3 (10.7%)	24 (33.3%)	0.04168
CT1 score of the operated lung			
a (*n*, %)	4 (14.3%)	22 (30.6%)	0.15810
b (*n*, %)	11 (39.3%)	32 (44.4%)	0.80810
c (*n*, %)	13 (46.4%)	18 (25.0%)	0.06583
Calcified nodules on CT1 scan			
Present (*n*, %)	7 (25.0%)	34 (51.5%)	0.04276
CT2 score of the operated lung			
a (*n*, %)	1 (3.6%)	25 (34.7%)	0.00334
b (*n*, %)	13 (46.4%)	38 (52.8%)	0.72820
c (*n*, %)	14 (50.0%)	9 (12.5%)	0.00019
Calcified nodules on CT2 scan			
Present (*n*, %)	6 (21.4%)	52 (72.2%)	0.00001
Number of calcified nodules on CT2 scan			
Mean ± SD	0.32 ± 0.669	1.69 ± 2.179	0.00005
CT1-CT2 score of the operated lung			
a-a (*n*, %)	1 (3.6%)	18 (25.0%)	0.03011
c-c (*n*, %)	11 (39.3%)	5 (6.9%)	0.00026
Number of peripheral nodules on CT2 scan			
Mean ± SD	3.46 ± 2.603	5.67 ± 5.487	0.07873
Number of central nodules on CT2 scan			
Mean ± SD	0.79 ± 0.995	1.43 ± 1.814	0.10790
Total number of nodules on CT2 scan			
Mean ± SD	4.3 ± 2.62	7.1 ± 6.61	0.05962
Number of nodules of < 3.0 mm on CT2 scan			
Mean ± SD	2.32 ± 1.847	3.61 ± 3.766	0.24840
Number of nodules of 3.0–5.0 mm on CT2 scan			
Mean ± SD	1.54 ± 1.732	2.36 ± 2.804	0.1116
Number of nodules of 5.1–10.0 mm on CT2 scan			
Mean ± SD	0.39 ± 0.685	0.74 ± 1.034	0.11380
Number of nodules of > 10.0 mm on CT2 scan			
Mean ± SD	0 ± 0	0.39 ± 0.815	0.00381

CT1: baseline computed tomography scan; CT2: preoperative computed tomography scan; HP: histopathological; A—at least one nodule of ≥ 10 mm or three or more nodules of ≥ 5 mm; a—single nodule of > 10 mm or more than one nodule of > 5 mm; b—solitary nodule of 5–10 mm or multiple nodules of 3–5 mm; c—solitary nodule of < 5 mm or several nodules of < 3 mm; SD: standard deviation; *n*: number. *p* < 0.05 is statistically significant

### Logistic regression model

#### Univariate analysis

The results of the univariate analysis of CT1 and CT2 variables as potential predictors of metastases at TT are provided in Table 6. The features of the CT1 scans, including score “A” for both lungs, score “a” for the operated lung and the presence of calcified nodules, as well as the features of the CT2 scans, such as score “a” or “b” for the operated lung, the number of calcified nodules and total number of nodules, were all associated with a higher risk of malignancy (HP status “1” or “2”). The odds ratio (OR) for score “a” of the operated lung on the CT2 scan was almost 40 (*p* = 0.000927).

The significance of the number of nodules of < 3.0 mm on the CT2 scan is due to the significance of the total number of nodules on this scan, because these variables show a strong positive correlation (*p* < 0.00001; *r* = 0.91).

**Table 6:** Potential predictors of metastases (univariate analysis)

Predictor	OR (95% CI)	*p* Value
CT1 score of both lungs		
A	4.17 (1.29, 18.73)	0.03064
B	Reference	
CT1 score of the operated lung		
a	3.97 (1.18, 16.09)	0.03500
b	2.10 (0.78, 5.75)	0.14100
c	Reference	
Calcified nodules on CT1 scan		
Present	3.06 (1.17, 8.63)	0.02740
Absent	Reference	
CT2 score of the operated lung		
a	38.89 (6.45, 757.85)	0.000927
b	4.55 (1.63, 13.44)	0.004611
c	Reference	
Number of calcified nodules on CT2 scan	3.64 (2.09, 7.46)	0.000057
Number of central nodules on CT2 scan	1.39 (1.00, 2.15)	0.088600
Total number of nodules on CT2 scan	1.18 (1.10, 1.29)	0.000063
Number of nodules of < 3.0 mm on CT2 scan	1.30 (1.14, 1.51)	0.000240
Number of nodules of 3.0–5.0 mm on CT2 scan	1.18 (0.96, 1.54)	0.1607
Number of nodules of 5.1–10.0 mm on CT2 scan	1.63 (0.94, 3.27)	0.1171
Number of nodules of > 10.0 mm on CT2 scan	exp (17.6)	0.9914

CT1: baseline computed tomography scan; CT2: preoperative computed tomography scan; A—at least one nodule of ≥ 10 mm or three or more nodules of ≥ 5 mm; B—scans not meeting score A criteria; a—single nodule of > 10 mm or more than one nodule of > 5 mm; b—solitary nodule of 5–10 mm or multiple nodules of 3–5 mm; c—solitary nodule of < 5 mm or several nodules of < 3 mm; OR: odds ratio; CI: confidence interval; exp: exponential function. *p* < 0.05 is statistically significant

#### Multivariate analysis

**Fig. 2:**
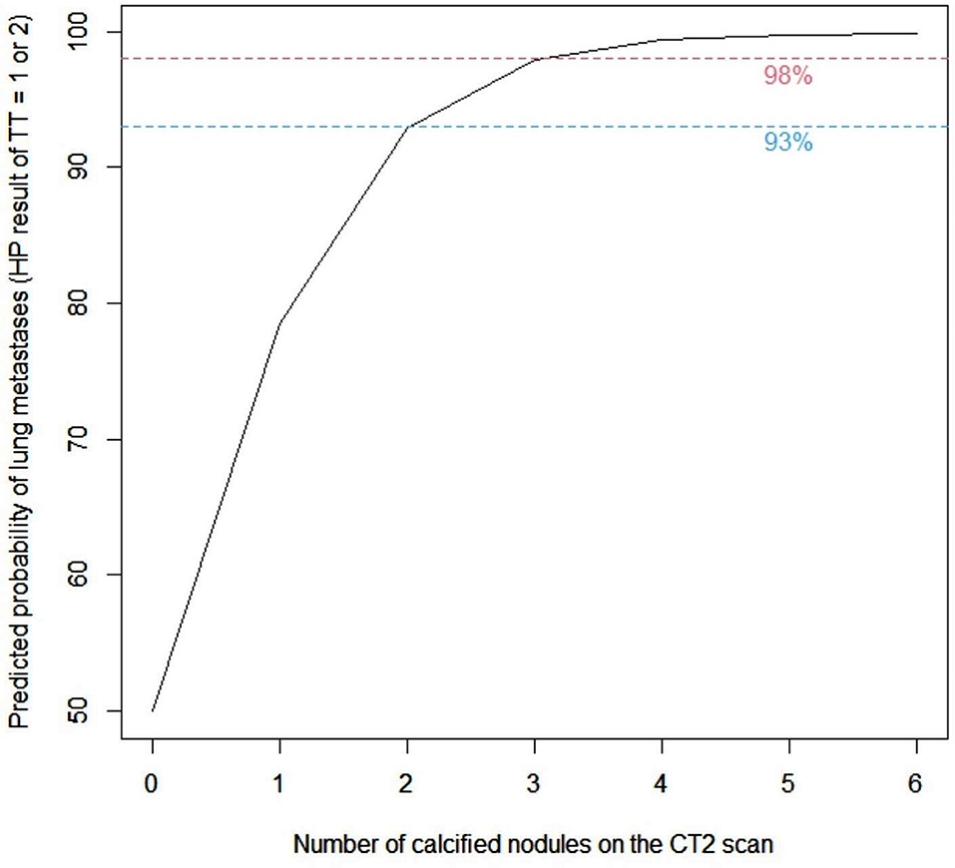
Predicted probability of lung metastases in patients with osteosarcoma (OS)

**Fig. 3:**
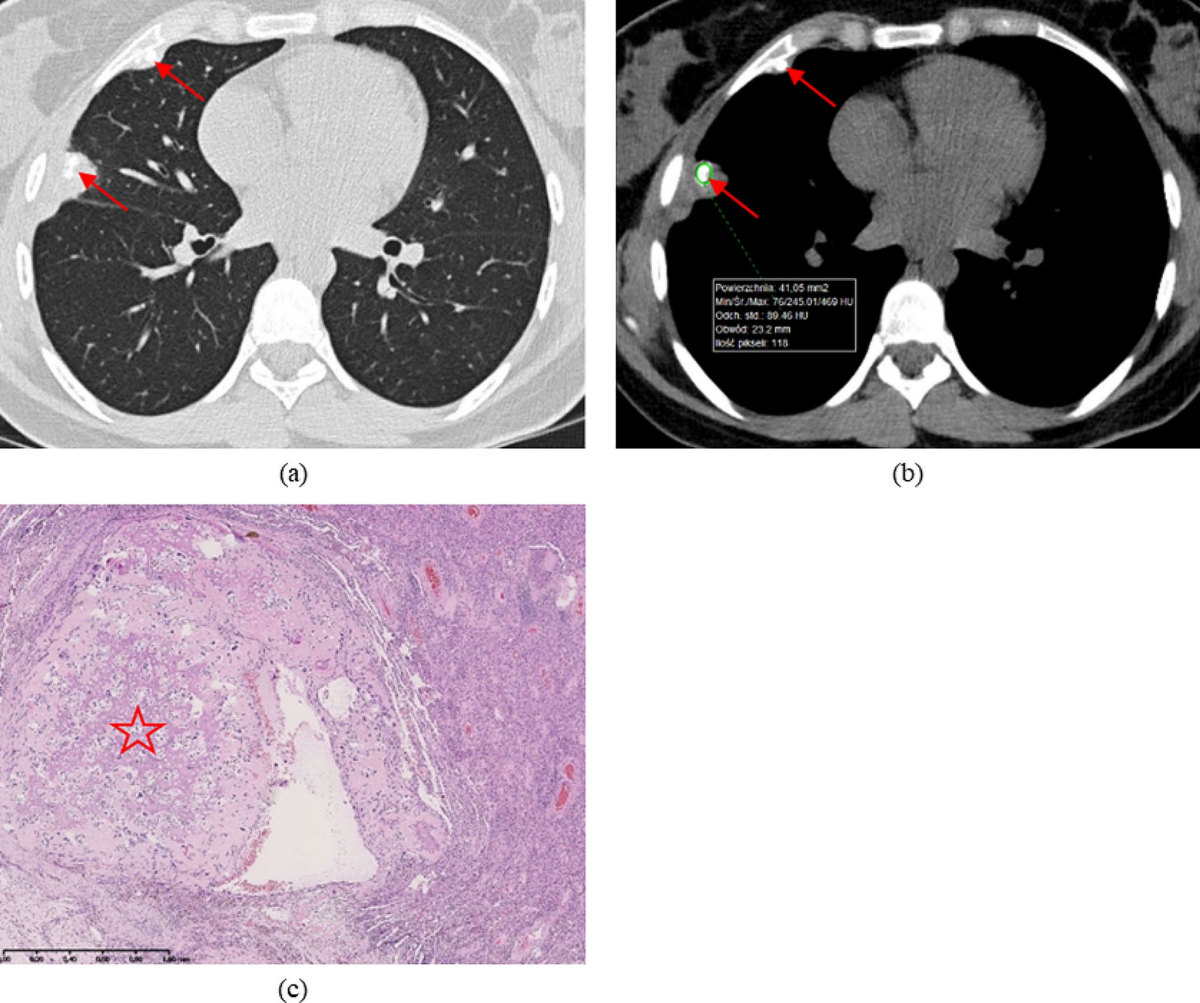
Late pulmonary recurrence of OS (11 years after the initial diagnosis). (**a**,**b**) Axial images from non-enhanced preoperative CT2 scan demonstrate two subpleural nodules with calcifications (arrows): (**a**) lung reconstruction; (**b**) mediastinal reconstruction with measurement from region of interest (ROI)—max. 469 Hounsfield units (HU). (**c**) HP slide of resected specimen at TT: haematoxylin and eosin (HE) staining; OS viable metastasis (conventional type) with partial regression after chemotherapy and central ossification (asterisk)

In the multivariate analysis that included all variables showing a univariate trend, the number of calcified nodules on the CT2 scan remained the only significant predictor of malignancy. With an increase in the number of these lesions by 1, the risk of metastases at TT (HP status “1” or “2”) increased more than threefold (Table 7). Using this model, it has been also found that the predicted probability of metastases in the operated lung was 93% for two calcified nodules on the CT2 scan, 98% for three such foci and almost 100% for four or more (Figs. 2 and 3).

**Table 7:** Predictors of metastases (multivariate analysis)

Predictor	OR (95% CI)	*p* Value
Number of calcified nodules on CT2 scan	3.42 (1.74, 7.99)	0.00155

CT2: preoperative computed tomography scan; OR: odds ratio; CI: confidence interval. *p* < 0.05 is statistically significant

### Decision tree model

**Fig. 4:**
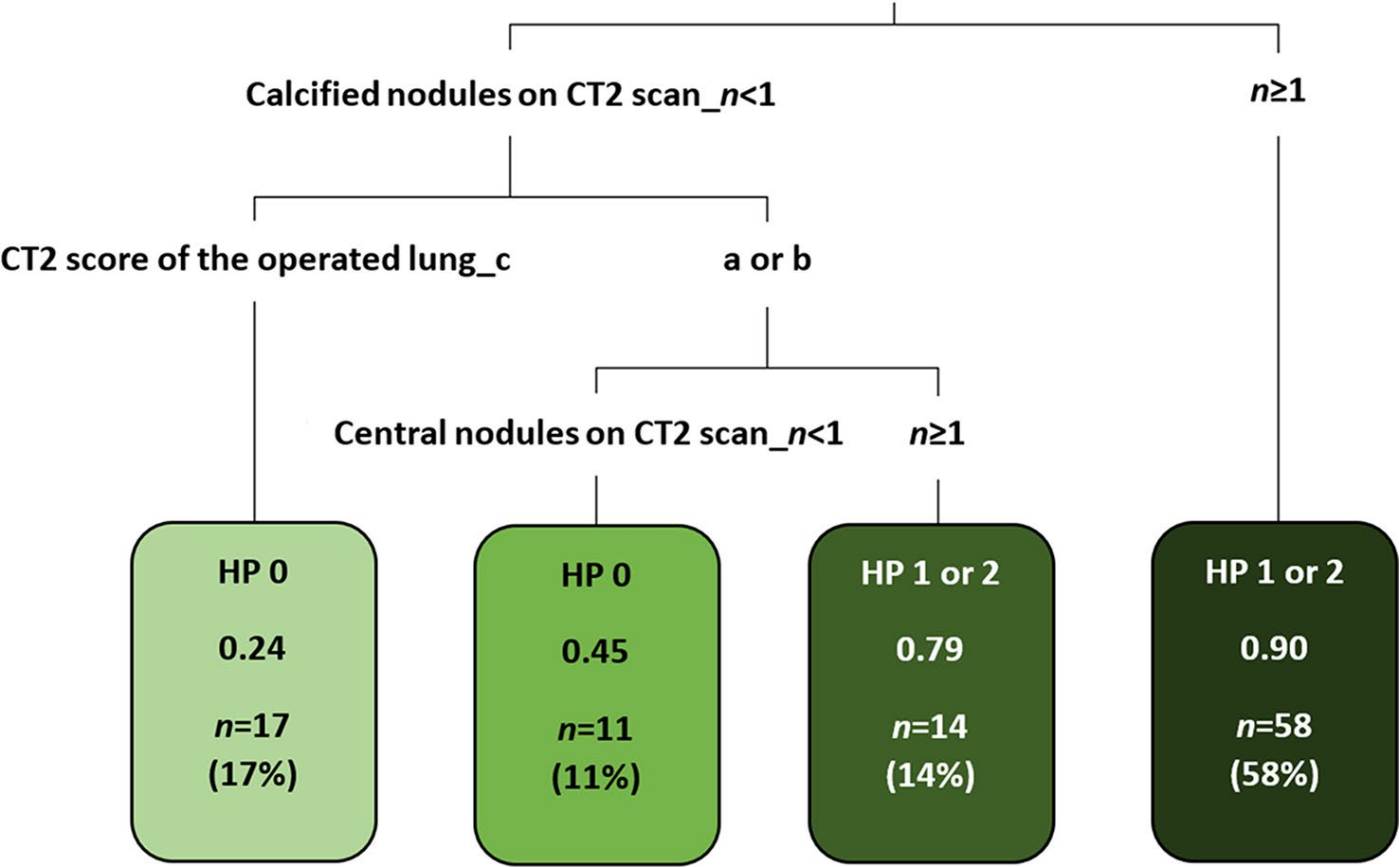
Schematic diagram of the decision tree model revealing predictors of HP status at TT

**Fig. 5:**
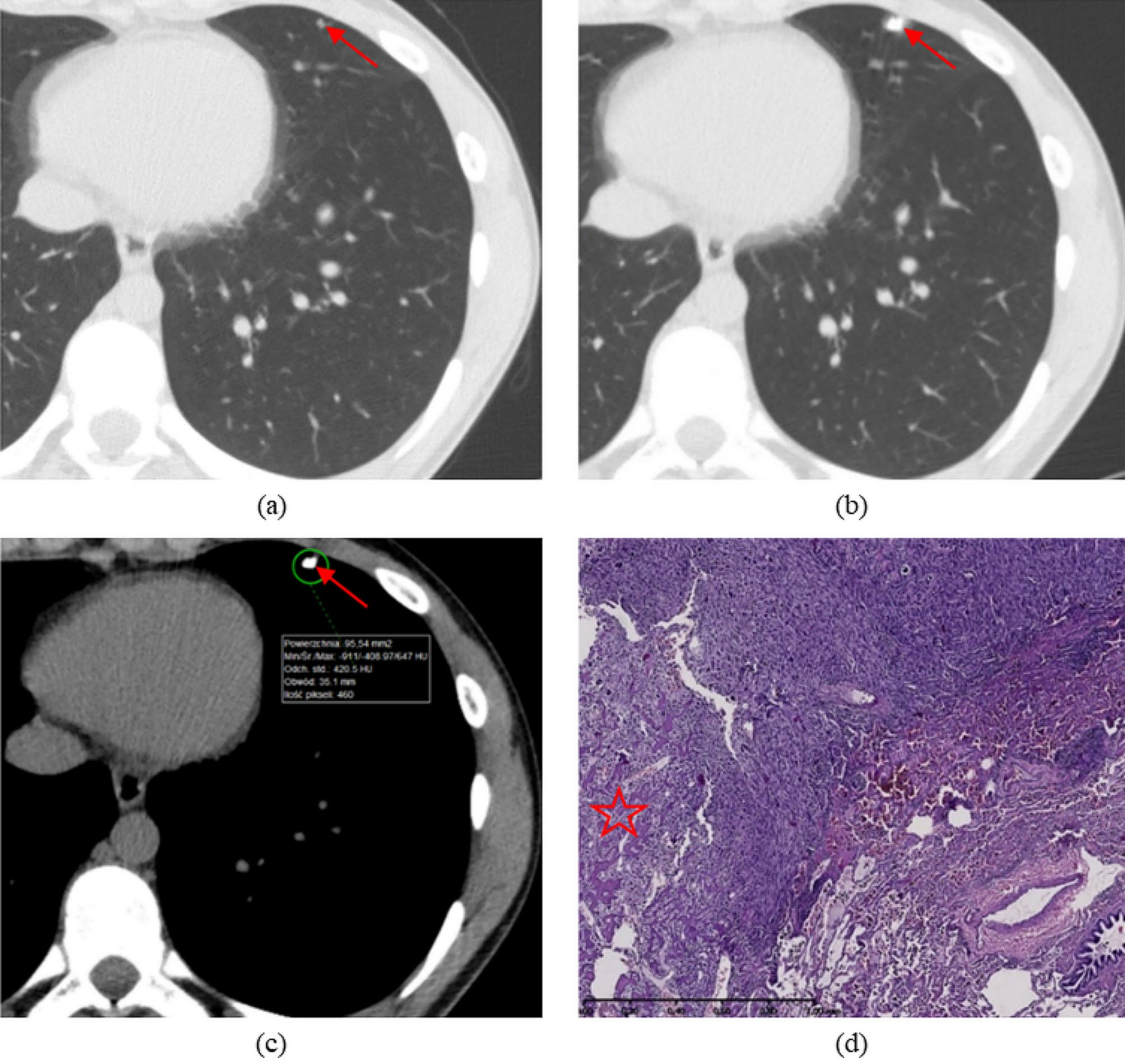
Pulmonary recurrence of OS 3.5 years after the initial diagnosis. (**a**–**c**) Axial images from non-enhanced CT scans demonstrate a solitary peripheral nodule (arrow): (**a**) baseline CT1 scan rated “c”, lung reconstruction; (**b**,**c**) preoperative CT2 scan 8 months later shows moderate progression (score “b”) but prominent calcification of the nodule; (**b**) lung reconstruction; (**c**) mediastinal reconstruction with measurement from ROI—max. 647 HU. (**d**) HP slide of resected specimen at TT: HE staining; OS viable metastasis (conventional type) with ossification (asterisk)

For TTs with calcified nodules on the CT2 scan (*n* = 58, 58%), the predicted (90% probability) status was malignant (HP result “1” or “2”). These TTs are represented by the rightmost leaf of the decision tree illustrated in Fig. 4. Moreover, for TTs without calcified nodules but with a CT2 score for the operated lung of “a” or “b” and at least one central nodule on this scan (*n* = 14, 14%), the predicted (79% probability) status was malignant. For TTs without calcified nodules, with a CT2 score of “a” or “b”, but without central nodules (*n* = 11, 11%), the predicted status was benign (HP result “0”), although as many as 45% of TTs with these features were confirmed malignant. For TTs without calcified nodules and with a CT2 score of “c” (*n* = 17, 17%), the predicted status was benign with a probability of 76%, while the remaining 24% (4/17) of TTs with these features were verified malignant, but all of them had at most foci of complete regression, i.e., nonviable metastases (leftmost leaf of the decision tree, Fig. 4). Figure 5 illustrates the most important predictors of malignancy revealed by the decision tree model: the presence of at least one calcified nodule on the CT2 scan and the rating of the operated lung on this scan.

### Random forest model

**Fig. 6:**
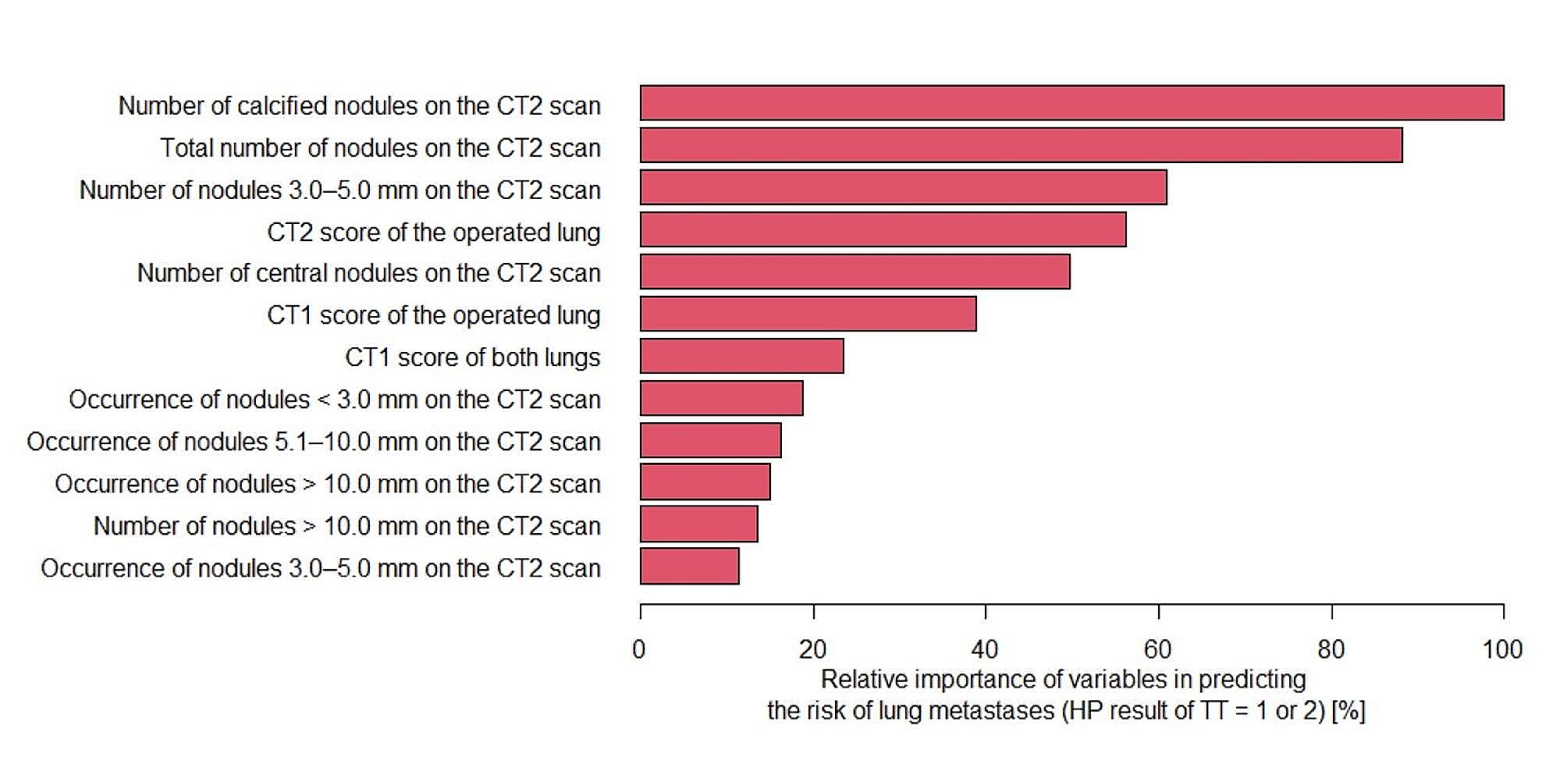
Ranking of predictors of TTs with lung metastases obtained from the random forest model

**Fig. 7:**
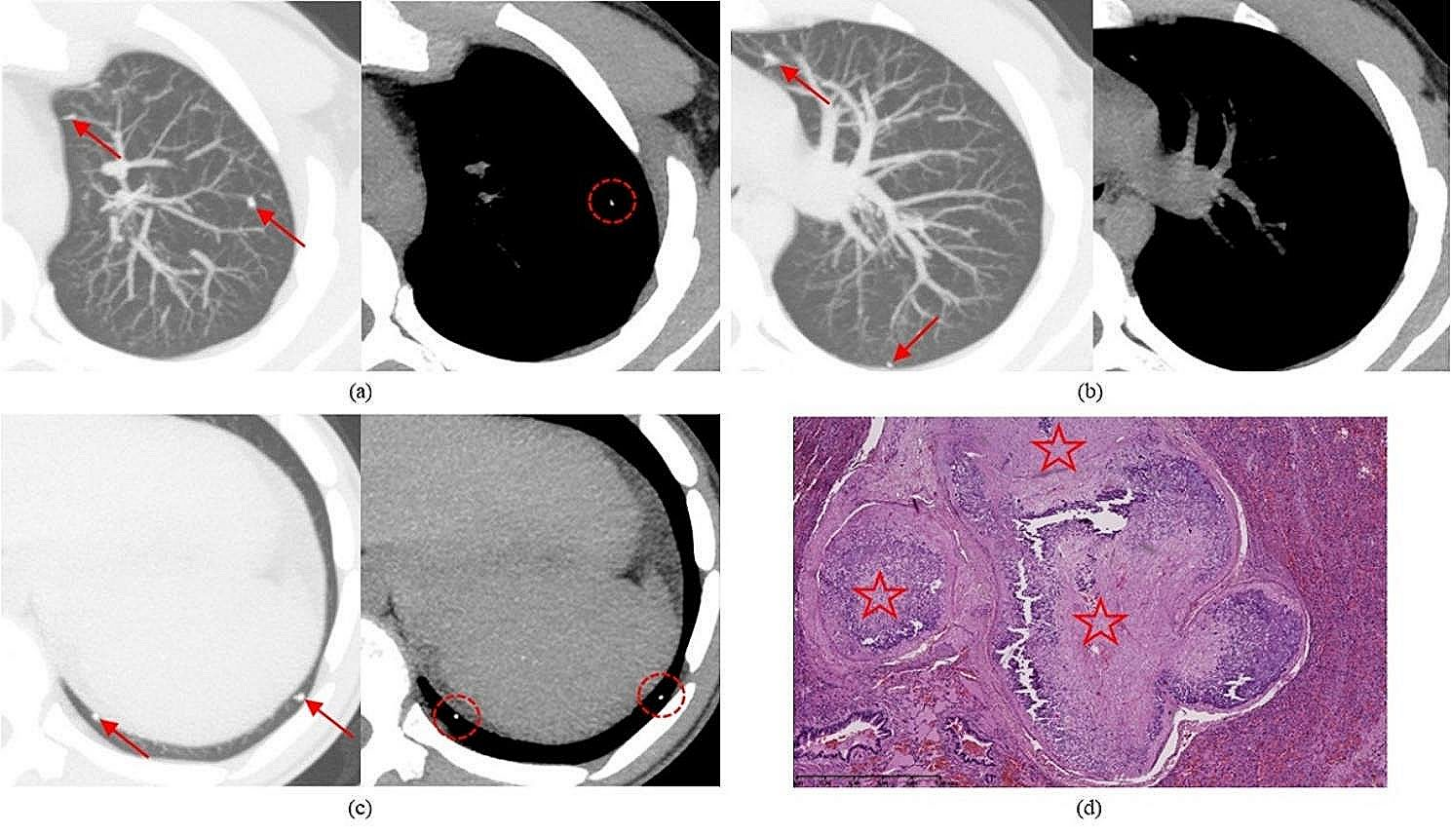
Pulmonary OS metastases at initial diagnosis. (**a**–**c**) Axial images from non-enhanced preoperative CT2 scan post-processed using the maximum intensity projection technique, displayed on split windows (**left**—lung reconstruction; **right**—mediastinal reconstruction), demonstrate multiple small nodules (arrows), with some arranged along vessels and some containing calcifications (ellipses). (**d**) HP slide of resected specimen at TT: HE staining; intravascular OS viable metastasis (conventional type) with ossifications (asterisks)

In the random forest model, the number of calcified nodules on the CT2 scan and the total number of nodules on this scan were at the top of the ranking of predictors of TTs with pulmonary metastases (Figs. 6 and 7). The CT2 rating of the operated lung was also positioned high on the list.

### Evaluation of predictive models

All three multivariate predictive models proved to have a relatively high sensitivity (range: 72–92%), PPV (range: 81–90%) and accuracy (range: 74–79%). The PPV of each model was 9 to 18 percentage points higher than the PPV of the qualification for TT performed at the time of treatment (Table 8), when only 72% of TTs confirmed viable or nonviable metastases (Table 4).

**Table 8:** Evaluation of multivariate predictive models and comparison with qualification for TT at the time of treatment

Predictive Model *	Sensitivity	Specificity	PPV	NPV	Accuracy
Logistic regression	0.722	0.786	0.900	0.520	0.740
Decision tree	0.875	0.679	0.875	0.679	0.790
Random forest	0.917	0.429	0.805	0.667	0.780
Qualification for TT at the time of treatment	-	-	0.720	-	-

* Refers to logistic regression, decision tree and random forest models; PPV: positive predictive value; NPV: negative predictive value; TT: thoracotomy

### Inter-observer reliability

Inter-reader agreement for the two radiologists reading the CT scans was almost perfect (κ > 0.8) or at least substantial (κ > 0.7) for the qualitative variables and excellent for all the quantitative variables (rc > 0.9).

## Discussion

Although CT is so far the best imaging modality for the detection of lung nodules as part of disease staging, it is often not sufficient for differentiating benign from malignant lesions [6, 11, 13–15]. Many authors have already attempted to find predictors of malignancy at the level of either the nodule or the patient, often concluding that there are no definite imaging criteria of lung metastases [13–15, 27]. In patients with OS, TT with manual exploration of the whole lung is the most recommended metastasectomy technique, while minimally invasive surgery focused on the resection of selected nodules is strongly discouraged [5, 22]. Taking into account the above, and also the retrospective nature of our study, which did not guarantee a reliable correlation of nodules detected on CT and resected at TT, we have chosen an analysis at TT level, instead of a nodule-for-nodule comparison of CT characteristics for pathology. Nevertheless, in order to properly rate the lungs, all 630 nodules detected on the preoperative CT2 scans were first assessed in detail. Our inclusion criteria allowed for a homogeneous cohort, composed of 64 paediatric patients with OS suspected of lung metastases, who underwent a total of 100 TTs after two scans per TT performed with the same MDCT scanner in most of the cases.

Complete metastasectomy is the best predictor of survival in patients with metastatic OS [25, 28, 30–32]. Even small microscopic deposits of regressing metastases may recur if the patient does not have a TT with the removal of all residual disease [18]. We therefore considered viable and nonviable metastases (the latter formed by a calcified osteoid matrix or by a matrix and necrotic material without sarcoma cells [33]), which are distinguishable on pathology, but not on CT, together in a single HP group “1 + 2” for most of our analyses.

The presence of pulmonary nodules in sarcoma patients does not always represent metastatic disease, and there are still far too many TTs confirming benign lesions only. This problem has been already highlighted by many authors, among them: McCarville et al. [14], Picci et al. [27], Brader et al. [13], Kayton et al. [33] and Ciccarese et al. [28]. In our study, there were 28 (28%) useless TTs.

To address the aim of our study, we constructed three various multivariate predictive models—the logistic regression model used by most authors [13, 14, 29, 34, 35] and two more—the decision tree and random forest models. This is a strength of our report, because to the best of our knowledge the latter two models have not been previously used in a similar cohort. We only found one recent (2021) report in which three machine learning models were used in an attempt to predict lung metastases in patients with OS, but the radiomic features were extracted from CT images of the local tumour [36].

All of our multivariate predictive models proved that calcified nodules on the preoperative CT2 scan—either the number of these foci (logistic regression and random forest models) or the mere fact of their occurrence (decision tree model)—have the strongest ability to predict pulmonary metastases. Furthermore, in the logistic regression model, it was found that the predicted probability of metastases in the operated lung is very high for two or more calcified nodules on the CT2 scan, reaching nearly 100% for four such foci. The correlation between HP status (malignant versus benign) and the calcification in OS patients is unique and the opposite of what is observed in the general population [13, 37]. The explanation of this fact is that the osteoid matrix produced by OS cells may mineralise and become both apparent upon imaging [2, 38] and readily palpated at TT as a small “grain of sand” [18]. Our findings are in agreement with those described by Brader et al. [13] and Ciccarese et al. [28]. Interestingly, the EURAMOS-1 [5] and COG [12] definition of metastases does not mention the calcification criterion. In our cohort, calcified nodules were more frequent on the CT2 scans (58% of TTs) than on the CT1 scans (41% of TTs). The broad histomorphological spectrum of OS, dependent on the predominant matrix [2], presumably influences imaging appearance on the baseline scan, while the higher incidence of calcified nodules on the preoperative scan is assumed to be induced by chemotherapy often administered in between the scans. Although both the presence of calcified nodules on the CT1 scan and the number of calcified nodules on the CT2 scan showed a univariate trend, only the latter finally proved to be a significant predictor of malignancy in the multivariate logistic regression model.

Moreover, two of the three multivariate predictive models revealed another significant predictor—the CT2 rating of the operated lung, dependent on the number and size of nodules. This variable is positioned on the second level of the decision tree and relatively high in the ranking obtained from the random forest model. Score “a” (single nodule of > 10 mm or more than one nodule of > 5 mm) and score “b” (solitary nodule of 5–10 mm or multiple nodules of 3–5 mm) are associated with an increased probability of malignancy, as revealed by the decision tree model. Analysing previous reports, we found that regarding the individual nodule size, most authors proposed a threshold for metastases of 5–6 mm [13, 15, 27–29, 39]. However, Ciccarese et al. [28], Kusma et al. [29] and Lautz et al. [39] admitted that nodules < 5 mm in diameter still have a reasonable likelihood of malignancy and therefore the proposed values cannot be considered as a strict rule. In turn, the definition provided by the EURAMOS-1 trial [5] and COG [12] concerns the patient (not the individual nodule). Their authors defined “certain” pulmonary metastases at presentation as three or more lesions which are ≥ 5 mm in maximum diameter or as a single lesion ≥ 1 cm. Although it is not straightforward to compare three different scoring systems (focused on the nodule or the patient, or the operated lung), our findings seem to be similar to the results of the cited papers, while the scores “a” and “b” criteria are closest to the EURAMOS-1 and COG definition.

In the random forest model, the total number of nodules on the CT2 scan was positioned very high (second place) in the ranking of predictors. A similar finding was described by Picci et al. [27], who claimed that in their cohort all patients with more than seven nodules were metastatic, as opposed to 31% of patients with only one nodule.

The impact of central nodules on the CT2 scan (positioned at the lowest level of the decision tree model and in fifth place in the ranking of the random forest model) seems to be less significant than that of the predictors discussed above. There are divergent reports in the literature. According to most authors, lung metastases are usually peripherally located [30, 35, 40], but Ciccarese et al. [28] found that they were equally located in the central and in the subpleural region, while benign lesions had a predilection for the subpleural region.

Interestingly, the most significant predictors of pulmonary metastases (calcified nodules, rating of the operated lung and total number of nodules) are all CT2 variables, suggesting that the preoperative CT scan is more important for qualifying patients with OS for metastasectomy than the baseline scan.

All three multivariate predictive models achieved a relatively high sensitivity, PPV and accuracy. If they were applied to our cohort at the time of treatment, the PPV would have increased from 72% to 81–90%. Using these models in the future may therefore contribute to reducing the percentage of unnecessary invasive thoracic procedures in children and adolescents with OS. The selected predictors of pulmonary metastases may serve as helpful tips during patient qualification for TT, rather than absolute indications, because the final decision about metastasectomy should always be made by an interdisciplinary team, taking into account the sine qua non conditions of TT, i.e., a locally controlled primary tumour, the absence of extrapulmonary metastases, completely resectable lung metastases and sufficient postoperative lung function. Further research with the use of applications based on predictive models, computer-aided detection on CT workstations or artificial intelligence will support the development of guidelines and optimise the process of selecting candidates for pulmonary metastasectomy through thoracotomy.

There are some limitations to our study. The first is the retrospective design of our single-centre trial. Our patient cohort was not very large, but it did not differ much from those found in the literature concerning this rare disease. Furthermore, the trial inclusion criteria entering patients at various stages of disease and treatment, as well as the compartmental evaluation method used to rate the lung, limited the assessment of change in the number and size of lesions over time.

## Conclusions

**Fig. 8:**
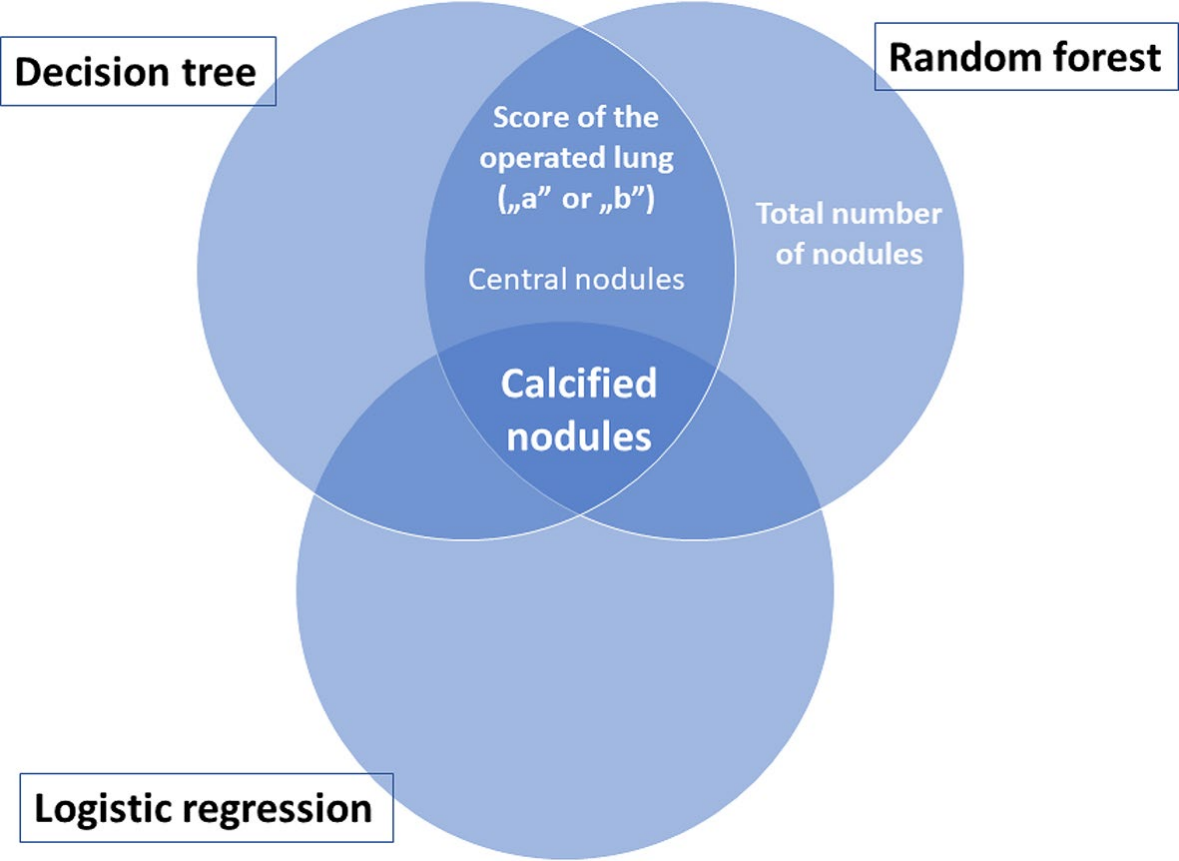
Venn diagram illustrating the most important predictors of pulmonary metastases on the preoperative CT scan. “a”—single nodule of > 10 mm or more than one nodule of > 5 mm; “b”—solitary nodule of 5–10 mm or multiple nodules of 3–5 mm

The three multivariate predictive models that were constructed and validated in this study revealed the most important predictors of lung metastases on the preoperative CT scan in children and adolescents with OS (Fig. 8), which can contribute to a decreased rate of useless thoracotomies in the future.

## Data Availability

The datasets used and/or analysed during the current study are available from the corresponding author on reasonable request.

## Abbreviations

CI: Confidence interval
COG: Children’s Oncology Group
CT: Computed tomography
CT1: Baseline CT scan
CT2: Preoperative CT scan
EURAMOS: European and American Osteosarcoma Study Group
HE: Haematoxylin and eosin
HU: Hounsfield units
HP: Histopathological
MDCT: Multi-row-detector computed tomography
MIP: Maximum intensity projection
MPR: Multiplanar reformatted reconstructions
NPV: Negative predictive value
OS: Osteosarcoma
PACS: Picture archiving and communication system
PPV: Positive predictive value
OR: Odds ratio
ROI: Region of interest
SD: Standard deviation
TT: Thoracotomy
